# Dissemination Dynamics of HIV-1 Subtype B Pandemic and Non-pandemic Lineages Circulating in Amazonas, Brazil

**DOI:** 10.3389/fmicb.2022.835443

**Published:** 2022-03-07

**Authors:** Ighor Arantes, Tiago Gräf, Paula Andrade, Yury Oliveira Chaves, Monick Lindenmeyer Guimarães, Gonzalo Bello

**Affiliations:** ^1^Laboratório de AIDS e Imunologia Molecular, Instituto Oswaldo Cruz, Fundação Oswaldo Cruz (FIOCRUZ), Rio de Janeiro, Brazil; ^2^Instituto Gonçalo Moniz, Fundação Oswaldo Cruz (FIOCRUZ), Salvador, Brazil; ^3^Laboratório de Diagnóstico e Controle de Doenças Infecciosas na Amazônia, Instituto Leônidas e Maria Deane, Fundação Oswaldo Cruz (FIOCRUZ), Manaus, Brazil

**Keywords:** HIV-1, subtype B, Brazil, Amazonas, phylodynamics

## Abstract

The HIV-1 epidemic in the Amazonas state, as in most of Brazil, is dominated by subtype B. The state, nonetheless, is singular for its significant co-circulation of the variants B_CAR_, which can mostly be found in the Caribbean region, and B_PAN_, a clade that emerged in the United States and aggregates almost the totality of subtype B infections world-wide. The Amazonian HIV-1 epidemic provides a unique scenario to compare the epidemic potential of B_PAN_ and B_CAR_ clades spreading in the same population. To reconstruct the spatiotemporal dynamic and demographic history of both subtype B lineages circulating in Amazonas, we analyzed 1,272 HIV-1 *pol* sequences sampled in that state between 2009 and 2018. Our phylogeographic analyses revealed that while most B_CAR_ infections resulted from a single successful founder event that took place in the Amazonas state around the late 1970s, most B_PAN_ infections resulted from the expansion of multiple clusters seeded in the state since the late 1980s. Our data support the existence of at least four large clusters of the pandemic form in Amazonas, two of them nested in Brazil’s largest known subtype B cluster (B_BR–I_), and two others resulting from new introductions detected here. The reconstruction of the demographic history of the most prevalent B_PAN_ (*n* = 4) and B_CAR_ (*n* = 1) clades identified in Amazonas revealed that all clades displayed a continuous expansion [effective reproductive number (*R*_e_) > 1] until most recent times. During the period of co-circulation from the late 1990s onward, the R_e_ of Amazonian B_PAN_ and B_CAR_ clusters behaved quite alike, fluctuating between 2.0 and 3.0. These findings support that the B_CAR_ and B_PAN_ variants circulating in the Brazilian state of Amazonas displayed different evolutionary histories, but similar epidemic trajectories and transmissibility over the last two decades, which is consistent with the notion that both subtype B variants display comparable epidemic potential. Our findings also revealed that despite significant advances in the treatment of HIV infections in the Amazonas state, B_CAR_ and B_PAN_ variants continue to expand and show no signs of the epidemic stabilization observed in other parts of the country.

## Introduction

The HIV-1 subtype B pandemic started when the ancestral virus arrived and first established itself in the Caribbean region during the mid-1960s (Gilbert et al., 2007). The subsequent subtype B spread generated a set of local clades, designated as B_CAR_, that remained mostly confined to the Caribbean region (Gilbert et al., 2007; Cabello et al., 2014). One of those viruses, however, migrated from the Caribbean to the United States around the late 1960s and established a pandemic clade, called B_PAN_, that was then disseminated worldwide (Worobey et al., 2016). The remarkable dissemination of the B_PAN_ clade was probably shaped by ecological, rather than virological factors (Gilbert et al., 2007; Arantes et al., 2020); but there are no studies comparing the epidemic potential of B_CAR_ and B_PAN_ clades co-circulating outside the Caribbean region.

The HIV-1 subtype B epidemic in Brazil is mainly driven by the B_PAN_ clade, with a few notable exceptions, like the Northern state of Amazonas, which is characterized by the co-circulation of the B_PAN_ (75%) and B_CAR_ (25%) variants at a high prevalence (Divino et al., 2016; Crispim et al., 2019; Gräf et al., 2021). Among regional HIV-1 epidemics in Brazil, the one in the state of Amazonas stands out as the second largest AIDS detection rate (34.8 cases per 100,000 inhabitants) in the country, well above the mean national rate (17.8 cases per 100,000 inhabitants) (Ministério da Saúde, 2020). Furthermore, despite important advances in HIV diagnosis and treatment, the Amazonas HIV/AIDS epidemic is not stabilized and has displayed a rising AIDS incidence over the last 10 years (Ministério da Saúde, 2020).

The Amazonian HIV-1 epidemic thus provides a great opportunity to compare the epidemic dynamics of the B_PAN_ and B_CAR_ clades spreading in the same population outside the Caribbean region. Previous studies identified four major B_PAN_ (B_PAN–BR–I_ to B_PAN–BR–IV_) and four major B_CAR_ (B_CAR–BR–I_ to B_CAR–BR–IV_) clusters circulating in Brazil (Mir et al., 2015; Divino et al., 2016). The lineage B_CAR–BR–I_ aggregates the majority (51%) of non-pandemic subtype B sequences from Brazil (Divino et al., 2016) and its most recent common ancestor (MRCA) could be traced back to Amazonas in the late 1970s, from a viral migration probably originated in the French Guiana (Divino et al., 2016; Arantes et al., 2019). The cluster composition and the evolutionary history of the B_PAN_ clade in the Amazonas state are currently unknown.

This work aims to characterize the spatiotemporal dynamics of the major B_PAN_ clusters circulating in the state of Amazonas and to compare the evolutionary and demographic history of major pandemic and non-pandemic subtype B lineages that drive the expanding HIV-1 subtype B epidemic in this Northern Brazilian state.

## Methodology

### HIV-1 Subtype B *pol* Sequence Dataset From Amazonas State

In this study, we used a total of 1,272 HIV-1 subtype B *pol* sequences (nucleotides 2,253–3,275 of reference strain HXB2) from Amazonas state sampled between 2009 and 2018 that were either available at the Los Alamos HIV Database^1^ by March 2021 or were recently published (Chaves et al., 2021; Gräf et al., 2021) and made available in GenBank^2^. Only one sequence per subject was selected. Sequences were aligned using the ClustalW program (Larkin et al., 2007) and all sites associated with major antiretroviral drug resistance in protease and reverse transcriptase were excluded.

### Phylogenetic Classification of Amazonian HIV-1 Subtype B Sequences

Amazonian HIV-1 subtype B sequences were initially classified as B_CAR_ or B_PAN_ and next within major B_CAR_ or B_PAN_ Brazilian clusters by using an evolutionary placement algorithm (EPA) available in RAxML v.8.0.0 (Stamatakis, 2014) for the rapid assignment of query sequences to edges of a reference phylogenetic tree under a maximum-likelihood (ML) model. This analysis allowed us to classify sequences within 10 different clades: B_CAR–BR–I_ to B_CAR–BR–IV_, B_PAN–BR–I_ to B_PAN–BR–IV_, other B_CAR_ lineages, and other B_PAN_ lineages.

### Selection of Brazilian and Global Reference HIV-1 Subtype B *Pol* Datasets

After the initial cluster assignment, the HIV-1 subtype B *pol* Amazonian sequences were aligned with different sub-sets of non-Amazonian subtype B *pol* reference sequences (covering nucleotides 2,253–3,260 relative to HXB2 genome). Amazonian subtype B sequences classified within major B_CAR_ (B_CAR–BR–I_ to B_CAR–BR–IV_) or B_PAN_ (B_PAN–BR–I_ to B_PAN–BR–IV_) Brazilian clusters were aligned with Brazilian sequences from other states that were previously classified within those major lineages (Mir et al., 2015; Divino et al., 2016). Amazonian subtype B sequences classified as “others B_CAR_ lineages” were aligned with sequences representative of the B_CAR_ diversity in the Caribbean region (*n* = 228) that were also described previously (Cabello et al., 2014; Mendoza et al., 2014). Finally, Amazonian subtype B sequences classified as “others B_PAN_ lineages” were aligned with: (i) one subset of closely related B_PAN_ sequences from Brazil (*n* = 687) selected from a large dataset of Brazilian sequences (*n* = 88,441) described previously (Gräf et al., 2021) and (ii) one subset of closely related B_PAN_ sequences from different countries (*n* = 1,700) selected from a large dataset of worldwide sampled sequences (*n* = 71,160) recovered from Los Alamos HIV Database. We used the basic local alignment search tool (BLAST)^3^ to select the 10 subtype B reference sequences (Brazilian and worldwide) with the highest similarity score (>95%) to each Amazonian subtype B sequence.

### Detection of Major Amazonian HIV-1 Subtype B Clades

Amazonian and non-Amazonian subtype B sequences were subject to ML phylogeographic analyses to identify the B_CAR_ and B_PAN_ sub-clusters that probably originated in Amazonas. ML phylogenetic trees were inferred with the PhyML program (Guindon et al., 2010) using an online web server (Guindon et al., 2005) under the GTR + I + Γ4 nucleotide substitution model, as selected by the jModelTest program (Posada, 2008), and the SPR branch-swapping algorithm of heuristic tree search. The reliability of the obtained tree topology was estimated with the approximate likelihood-ratio test (aLRT) (Anisimova and Gascuel, 2006) based on the Shimodaira-Hasegawa-like procedure. Trees were rooted using subtype D sequences and visualized using the FigTree v1.4.0 software (Rambaut, 2009). The ML trees were employed for the ancestral character state reconstruction (ACR) of epidemic locations with PastML (Ishikawa et al., 2019), using the maximum likelihood Joint (Pupko et al., 2000) and marginal posterior probabilities approximation (MPPA) methods with an F81-like model. Beyond Amazonas, remaining Brazilian states were aggregated in five discrete locations according to their geographic regions and sequences from other countries were aggregated in a single “non-Brazilian” location. Amazonian B_CAR_ and B_PAN_ clades were defined as those monophyletic clusters with high support (aLRT ≥ 0.85) that were mostly composed of sequences from Amazonas (>85%) and displayed Amazonas as the most probable (*P* ≥ 0.85) state location of its MRCA. Amazonian clades were further subdivided according to size into large (*n* > 30), medium (*n* = 10–30), and small (*n* = 2–9) clades.

### Phylodynamic Analysis

The study of epidemiological and evolutionary parameters of major B_CAR_ and B_PANDEMIC_ clusters from Amazonas was done by Bayesian inference using coalescent and birth-death tree priors implemented, respectively, in BEAST v1.10 (Drummond et al., 2002; Suchard et al., 2018) with BEAGLE (Suchard and Rambaut, 2009) to improve run-time, and in BEAST 2.6 (Bouckaert et al., 2019) software packages. Dated phylogenies were inferred with the flexible Bayesian skyline coalescent model (Drummond, 2005). Changes across time in their effective sample size (N_e_) were estimated using the coalescent Bayesian Skygrid (BSKG) model (Gill et al., 2012), and in their effective reproductive number (R_e_), using the Birth-death Skyline (BDSKY) model (Stadler et al., 2012). For BDSKY, the sampling rate (δ) was set to zero for the period before the oldest sample and estimated afterward. The R_e_ was modeled in a piecewise manner in equidistant intervals from the most recent sample up to the root of the tree with a lognormal prior (mean = 0; standard deviation = 1), and the becoming non-infectious rate with a lognormal prior (mean = 0.25; standard deviation = 0.5). All Bayesian MCMC analyses were performed using the GTR + I + Γ4 nucleotide substitution model, and a relaxed uncorrelated lognormal molecular clock model (Drummond et al., 2006) with a uniform prior distribution on the substitution rate that encompasses mean values previously estimated for the subtype B *pol* gene (2.0–3.0 × 10^–3^ subst./site/year) (Hue et al., 2005; Zehender et al., 2010; Chen et al., 2011; Mendoza et al., 2014; Bello et al., 2018). MCMC chains were run for 50–100 × 10^6^ generations and convergence and uncertainty of parameter estimates were assessed by calculating the effective sample size (ESS) and 95% highest probability density (HPD) values, respectively, after excluding the initial 10% of each run with Tracer v1.7.1 (Rambaut et al., 2018). The convergence of parameters was considered when ESS ≥ 200.

### Statistical Analysis

Demographic information of age group and gender of individuals with samples included in the present study were compared using Pearson’s chi-squared test as implemented in R version 3.6.3 (R Core Team, 2018), with 10,000 replicates. The false discovery rate (FDR) method was used to correct for multiple hypothesis testing and to reduce false positives. Statistical significance was defined as *p*-values <0.05.

## Results

**FIGURE 1:**
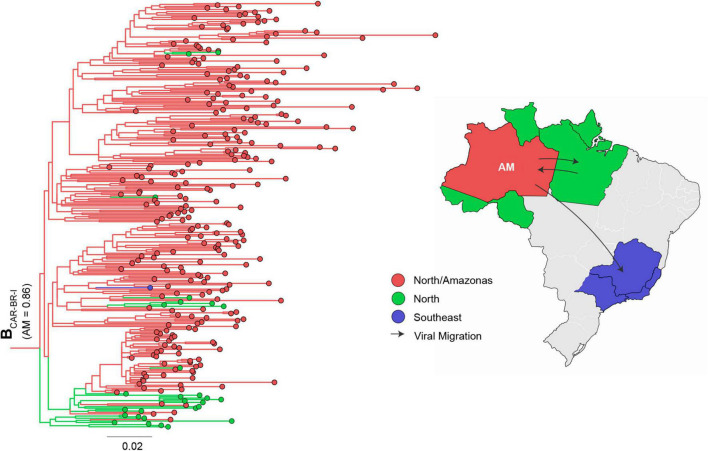
ML phylogeographic analysis of major cluster B_CAR–BR–I_ disseminated in Brazil. A total of 296 B_CAR–BR–I_ Brazilian sequences sampled in Amazonas (*n* = 267) and other states (*n* = 29) were analyzed. The number in parenthesis indicates the inferred marginal probability that the clade ancestor was located in the Amazonas state. The location of taxonomic units at internal nodes across the ML tree was reconstructed and represented according to the color scheme shown in the map. Outside Amazonas state, other Brazilian units were aggregated according to the country region. AM, Amazonas; N, North; SE, Southeast. The tree was rooted using HIV-1 subtype D reference sequences (not shown). The branch lengths are drawn to scale with the bar at the bottom indicating nucleotide substitutions per site. Major migrations of viral clade B_CAR–BR–I_ are represented in the map.

**FIGURE 2:**
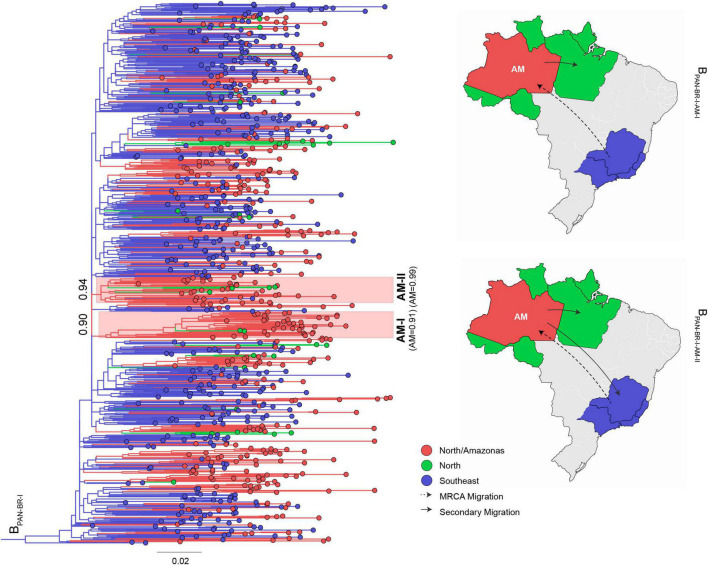
ML phylogeographic analysis of major B_PAN–BR–I_ cluster disseminated in Brazil. A total of 844 B_PAN–BR–I_ Brazilian sequences sampled in Amazonas (*n* = 360) and other states from the Northern and Southeastern regions (*n* = 484) were analyzed together. We selected sequences from Northern and Southeastern states because were the regions more strongly connected with the Amazonas state. Major Amazonian clades B_PAN–BR–I–AM–I_ and B_PAN–BR–I–AM–II_ are indicated by colored shaded boxes along with cluster aLRT support (number at basal branch) and the inferred marginal probability that cluster ancestor was located in Amazonas state (in parentheses). The location of taxonomic units at internal nodes across the ML tree was reconstructed and represented according to the color scheme shown in the map. Sequences were grouped in three discrete locations: Amazonas state (AM), Northern region (N), and Southeastern region (SE). The tree was rooted using HIV-1 subtype D reference sequences (not shown). The branch lengths are drawn to scale with the bar at the bottom indicating nucleotide substitutions per site. Major migrations of viral clades B_PAN–BR–I–AM–I_ and B_PAN–BR–I–AM–II_ are represented in the map.

**FIGURE 3:**
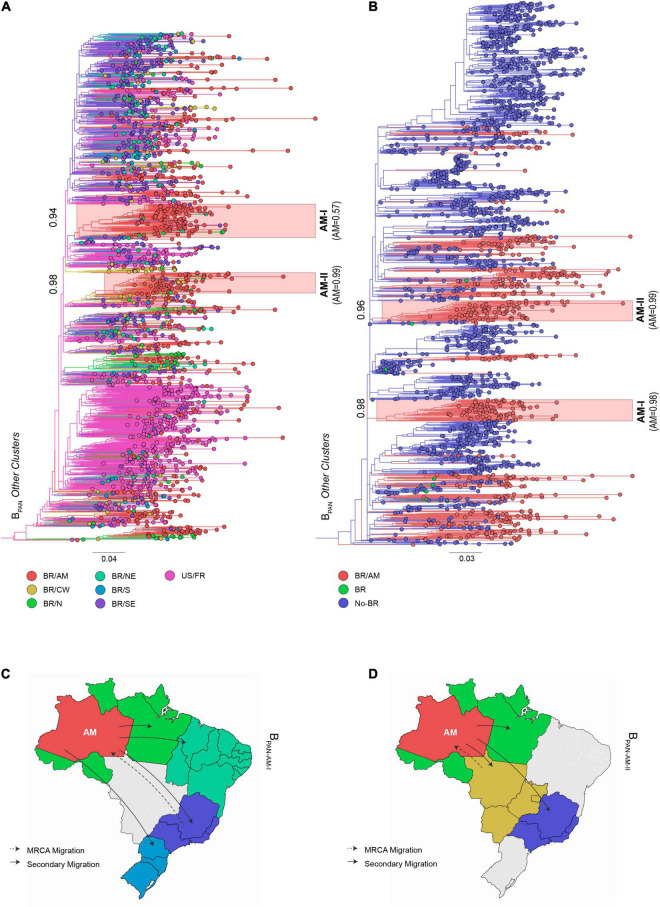
ML phylogeographic analysis of HIV-1 B_PAN_ Amazonian sequences that branched outside major Brazilian clades. A total of 530 B_PAN_ sequences sampled in Amazonas were aligned with: **(A)** Closely related B_PAN_ sequences sampled in other Brazilian states (*n* = 687) plus reference B_PAN_ sequences from the United States and France (*n* = 498), and **(B)** closely related B_PAN_ sequences sampled worldwide (*n* = 1,683). Major Amazonian clades B_PAN–AM–I_ and B_PAN–AM–II_ are indicated by colored shaded boxes along with cluster aLRT support (number at basal branch) and the inferred marginal probability that cluster ancestor was located in Amazonas state (in parenthesis). The location of taxonomic units at internal nodes across the ML tree was reconstructed and represented according to the color scheme shown in the map. Sequences were grouped in seven **(A)** and three **(B)** discrete locations: Amazonas state (AM), Central-Western region (CW), Northern region (N); Northeastern region (NE), Southeastern region (SE), Southern region (S), United States/France (US/FR), other Brazilian states (BR), and non-Brazilian (Non-BR). Tree was rooted using HIV-1 subtype D reference sequences (not shown). The branch lengths are drawn to scale with the bar at the bottom indicating nucleotide substitutions per site. Major migrations of viral clades **(C)** B_PAN–AM–I_ and **(D)** B_PAN– AM–II_ are represented in the maps.

The 1,272 HIV-1 subtype B *pol* sequences from Amazonas were assigned to either B_CAR_ (23%) or B_PAN_ (77%) lineages (Table 1). The sub-lineage assignment reveals that most B_CAR_ Amazonian sequences belong to the major Brazilian clade B_CAR–BR–I_ (89%), while the remaining sequences were classified within clades B_CAR–BR–II_ (1%), B_CAR–BR–III_ (1%), or branched outside known Brazilian clades (8%) (Table 1). Although a high proportion of B_PAN_ Amazonian sequences also branched within major Brazilian B_PAN_ clades (45%), particularly the B_PAN–BR–I_ (37%), most pandemic sequences (55%) branched outside known countrywide Brazilian clades (Table 1). To identify the major subtype clades circulating in Amazonas, we next conducted independent ML phylogeographic analyses for: (i) sequences that branched within major Brazilian B_CAR_ or B_PAN_ clades, by combining Brazilian sequences sampled in Amazonas and other states, and (ii) sequences that branched outside major Brazilian clades, by combining Amazonian sequences with either B_CAR_ sequences of Caribbean origin or B_PAN_ sequences sampled in Brazil and worldwide. The ML phylogeographic analyses confirm multiple introductions of B_CAR_ (*n* = 18) and B_PAN_ (*n* = 291) variants into the Amazonas state (Supplementary Table 1). The major B_CAR_ founder event resulted in the B_CAR–BR–I_ clade (Figure 1) while the remaining B_CAR_ sequences were distributed among a few local clusters of small size (2–9 sequences, 7%) or singletons (4%) (Supplementary Figures 1A–C). A few B_PAN_ introductions (*n* = 4) originated highly supported (aLRT > 0.85) Amazonian B_PAN_ clades of large size that were mostly composed by sequences from Amazonas (>85%) and most probably arose in Amazonas (*P* > 0.90). Two major clusters, B_PAN–BR–I–AM–I_ (*n* = 39) and B_PAN–BR–I–AM–II_ (*n* = 35), were nested within the large Brazilian clade B_PAN–BR–I_ (Figure 2). The other two clusters, B_PAN–AM–I_ (*n* = 86) and B_PAN–AM–II_ (*n* = 60), branched outside the major B_PAN_ Brazilian clades (Figure 3). Because the phylogenetic placement of some Amazonian basal sequences in clusters B_PAN–AM–I_ and B_PAN–AM–II_ changed according to the reference (Brazilian or worldwide) dataset, we define the final size of those clusters by the monophyletic groups supported by both analyses. The four major Amazonian B_PAN_ clades, together, comprise 23% of all B_PAN_ Amazonian sequences analyzed and the remaining sequences branched within Amazonian clades of medium (24%) or small (35%) size or appeared as singletons that branched with non-Amazonian sequences (18%) (Figures 2, 3 and Supplementary Figures 1D–F).

**TABLE 1:** Lineage classification of HIV-1 subtype B *pol* sequences from Amazonas state.

Lineage	Sub-lineage	*N* (%)	Sampling range
B_PAN_	B_PAN–BR–I_	360 (37%)	2009–2018
	B_PAN–BR–II_	38 (4%)	
	B_PAN–BR–III_	23 (2%)	
	B_PAN–BR–IV_	23 (2%)	
	Others	530 (55%)	
	**Total**	**974 (100%)**	
B_CAR_	B_CAR–BR–I_	267 (90%)	2009–2018
	B_CAR–BR–II_	2 (1%)	2016–2017
	B_CAR–BR–IV_	4 (1%)	2015–2017
	Others	25 (8%)	2009–2018
	**Total**	**298 (100%)**	

*The table details the distribution of 1,272 HIV-1 subtype B pol sequences (nucleotides 2,253–3,275 of reference strain HXB2) from Brazil’s Amazonas state across the known clusterization profile of the pandemic (B_PAN_, n = 974, 77%) and non-pandemic (B_CAR_, n = 298, 23%) forms. For each cluster, its absolute and relative frequency of samples from Amazonas state, as well as their distribution in time, are indicated. The class “Other” aggregates sequences not clustered among established major Brazilian clusters.*

**FIGURE 4:**
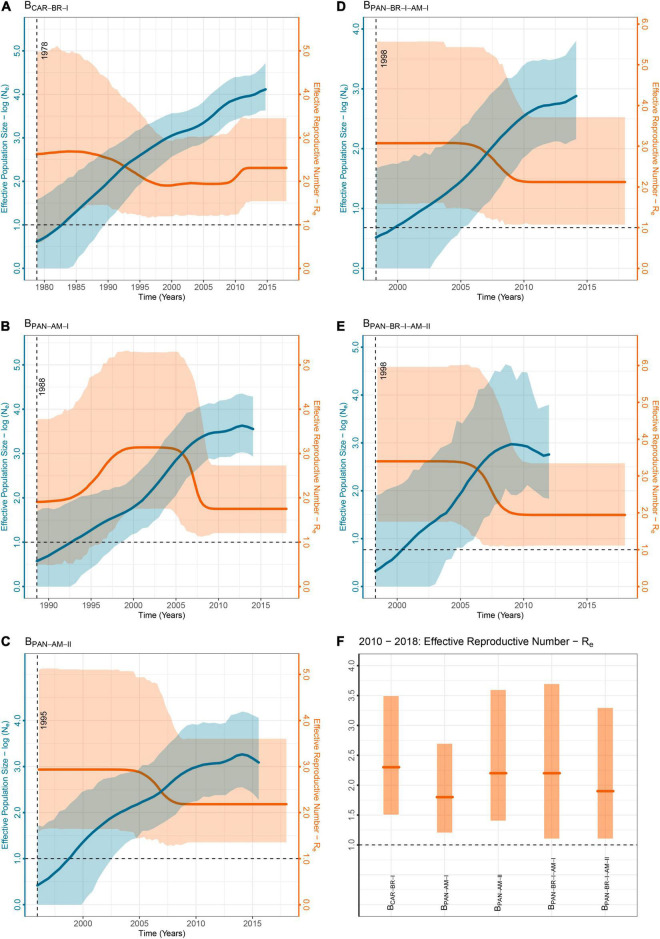
Demographic history of subtype B cluster in Brazil’s Amazonas state. Each plot **(A–E)** details the demographic history of one subtype B large cluster (*n* ≥ 30) in Amazonas from the pandemic (B_PAN_) and non-pandemic (B_CAR_) forms. The graphs exhibit their effective number of HIV-1 infections under the Bayesian Skygrid (BSKG) model in blue (N_e_, *y*-left-axis), and their effective reproductive number under the Birth-death Skyline (BDSKY) model in orange (N_e_, *y*-right-axis). For both parameters are indicated their median (solid lines) and 95% HPD intervals (pale areas) estimates. A dashed vertical line indicates the TMRCA of the clades, accompanied by its median value. The last graph **(F)** compares the R_e_ obtained for the five clusters in the last period of analysis (2010–2018). For each cluster, its median R_e_ (solid line) and 95% HPD interval (pale area) inferred values are represented.

To study in more detail the evolutionary and demographic history of lineages B_CAR_ and B_PAN_ spreading in Amazonas, we selected the five major clades that display both local epidemic importance – as, combined, they comprise 38% of HIV-1 subtype B infections in the state – and adequate sample sizes to give reliable demographic estimates. Time-scaled trees were reconstructed using a Bayesian coalescent model with an informative clock rate prior due to the weak temporal structure of Amazonian subtype B *pol* datasets (Supplementary Figure 2). Posterior estimates, that were, as expected, largely influenced by the selected clock rate prior, traced the median T_MRCA_ of major Amazonian clades to the late 1970s for B_CAR–BR–I_, the late 1980s for B_PAN–AM–I_, the mid-1990s for B_PAN–AM–II_, and the late 1990s for B_PAN–BR–I–AM–I_ and B_PAN–BR–I–AM–II_ (Table 2). These findings support that the major B_CAR_ clade was successfully spreading in Amazonas for about 10 years before the emergence of the major B_PAN_ clades, which may explain the singular high prevalence of non-pandemic subtype B variants in Amazonas with respect to most other Brazilian states. The BSKG model supports that the N_e_ of lineages B_CAR–BR–I_ and B_PAN–BR–I–AM–I_ steadily increased until recent years, while the N_e_ of lineages B_PAN–AM–I_, B_PAN–AM–II_, and B_PAN–BR–I–AM–II_ increased until the late-2000s, but then stabilized in more recent years (Figures 4A–E). The temporal trajectories of the R_e_ estimated using the Bayesian BDSKY model, however, support that all major Amazonian HIV-1 subtype B clades continuously expanded (median *R*_e_ > 1) over all the studied period, with some temporal fluctuations in the rate of expansion (Figures 4A–E). The clade B_CAR–BR–I_ reached the highest median *R*_e_ (2.5–2.6) between the late 1970s and the early 1990s, while the B_PAN_ clades reached the highest median *R*_e_ (2.9–3.4) between the mid-1990s and the mid-2000s (Table 2). Despite those differences in the early phase of spread, all major Amazonian subtype B clades converge to the roughly similar median growth rate (*R*_e_ = 1.6–2.3) at the most recent time period analyzed (2010–2018), with no evidence of recent epidemic stabilization (*R*_e_ > 1) (Figure 4F).

**TABLE 2:** Bayesian estimates of evolutionary and demographic parameters of major HIV-1 subtype B clades originated in the Amazonas state.

Clade	*N*	T_MRCA_*^a^* (95% HPD)	R_e_*^b^* (95% HPD)	
B_CAR–BR–I_	267	1978 (1970–1987)	1978–1985	2.6 (0.2–4.6)
			1986–1993	2.5 (1.5–4.2)
			1994–2001	1.9 (1.2–3.0)
			2002–2009	1.9 (1.2–3.0)
			2010–2018	2.3 (1.5–3.5)
B_PAN–AM–I_	86	1988 (1981–1994)	1988–1997	2.0 (0.5–4.1)
			1998–2007	2.9 (1.6–4.8)
			2008–2018	1.6 (1.1–2.6)
B_PAN–AM–II_	60	1995 (1990–2000)	1995–2006	2.9 (1.6–5.4)
			2007–2018	2.2 (1.4–3.6)
B_PAN–BR–I–AM–I_	39	1998 (1992–2002)	1998–2007	3.1 (1.6–5.6)
			2008–2018	2.1 (1.1–3.7)
B_PAN–BR–I–AM–II_	35	1998 (1992–2003)	1998–2007	3.4 (1.7–5.9)
			2008–2018	1.9 (1.1–3.3)

*The table details the evolutionary and epidemiological parameters of the major HIV-1 subtype B clusters of pandemic (B_PAN_) and non-pandemic (B_CAR_) forms circulating in Brazil’s Amazonas state.*

*^a^Median value and 95% HPD interval of the time to the most recent common ancestor (T_MRCA_).*

*^b^Median value and 95% HPD interval of the effective reproductive number (R_e_) inferred in a bird-death statistical model.*

## Discussion

Previous studies demonstrate that the expanding HIV-1 subtype B epidemic in the Northern Brazilian state of Amazonas was driven by both pandemic (B_PAN_) and non-pandemic (B_CAR_) viral variants (Cabello et al., 2015; Divino et al., 2016; Arantes et al., 2019; Crispim et al., 2019; Chaves et al., 2021; Gräf et al., 2021), thus creating a great opportunity to compare the epidemic dynamics of both subtype B forms spreading in the same population. This study revealed that the B_PAN_ and B_CAR_ epidemics in Amazonas have been shaped by different evolutionary histories, but displayed very similar transmissibility and expansion dynamics at most recent times.

Our analysis confirmed that variants B_CAR_ and B_PAN_ were introduced multiple times in the Amazonas state, although the estimated number of B_PAN_ introductions was 16 times higher than that of B_CAR_. The founder event that originated the clade B_CAR–BR–I_ occurred in the late 1970s (Divino et al., 2016; Arantes et al., 2019) and gave rise to 89% of B_CAR_ and 19% of total subtype B infections in Amazonas. In sharp contrast, most B_PAN_ sequences from Amazonas branched into multiple state-specific clusters of medium/small size (57%) or appeared as unclustered infections (19%). The four largest B_PAN_ Amazonian clades identified probably arose between the late 1980s and late 1990s and, together, comprise 23% of the B_PAN_ and 17% of all subtype B infections in the state. These findings support that the early introduction (late 1970s) of the B_CAR–BR–I_ ancestor in Amazonas from neighboring Caribbean countries probably drove its successful establishment and wide dissemination in the state. Although the B_PAN_ strains arrived in Amazonas later, they reached a high prevalence because they were introduced at much higher numbers and spread through more transmission networks than B_CAR_ strains.

The AIDS detection rate increased ∼10% in the Amazonas state between 2009 and 2019 (Ministério da Saúde, 2020). This finding is consistent with our BDSKY analyses that support a continuous expansion (*R*_e_ > 1) of major B_CAR_ and B_PAN_ Amazonian clades over all the studied period. The BSKG model indicates a recent stabilization of some Amazonian B_PAN_ clades since the late 2000s, and a previous study conducted by our group also indicated a recent epidemic stabilization of the clade B_CAR–BR–I_ since the late 2000s (Arantes et al., 2019). Although the median estimated R_e_ of the B_CAR_ and B_PAN_ Amazonian clades was somewhat lower between 2010 and 2018 (1.6–2.3) than during the previous decades (2.5–3.4), we found no solid evidence of epidemic stabilization or reduction in the BDSKY analyses. A previous study pointed out that the BSKG model requires strongly informative data to prevent erroneous estimates of N_e_ stabilization (Volz and Didelot, 2018). Thus, we hypothesize that the much larger number of recent (2009–2018) B_CAR–BR–I_ sequences used in the present study (*n* = 267) compared to the previous one (*n* = 45) allowed us to obtain a more accurate demographic reconstruction of the epidemic pattern in the last two decades.

It is interesting to note that the B_CAR_ (2.5–2.6) and B_PAN_ (2.9–3.4) Amazonian clades reached similar highest median *R*_e_ values. Furthermore, the highest median R_e_ estimated here using a birth-death approach was comparable to the previous ones estimated for the B_CAR–BR–I_ clade (3.8) using a coalescent-based approach (Arantes et al., 2019), but lower than those estimated for major B_PAN_ Brazilian lineages spreading in the Southeastern region (5.0–7.9) (Mir et al., 2015). These findings support that differences in the spreading dynamics of subtype B lineages may reflect discrepancies in the connectivity of underlying transmission networks across different Brazilian states/regions, rather than intrinsic differences in viral transmissibility. A preliminary analysis of the available demographic data (sex and age) of HIV-infected subjects from Amazonas revealed no significant differences between major B_CAR_ and B_PAN_ clades (Supplementary Table 2), supporting that both viral lineages are possibly spreading through networks with similar epidemiological properties. This observation is also consistent with a previous study that revealed comparable epidemic growth rates of B_CAR_ and B_PAN_ lineages circulating in the French Guiana (Bello et al., 2018).

Our study has some limitations. First, inferences about potential sources, total number of viral introductions, and local clade size in Amazonas were limited by both the incomplete sampling of local population and the limited number of non-Amazonian reference sequences included in each ML phylogenetic analysis as revealed by the variable phylogenetic and phylogeographic placement of some Amazonian sequences that branched basal to each local clade. The bulk of B_CAR_ and B_PAN_ sequences that compose each major Amazonian clade, however, remained constant across analyses, and our major phylogeographic conclusions were robust to sampling bias. Second, time-scale reconstructions were largely influenced by the selected clock rate prior due to the weak temporal structure of Amazonian HIV-1 subtype B *pol* datasets. Despite this, the T_MRCA_ here obtained were fully consistent with the overall time-scale of dissemination of the HIV-1 B_CAR_ and B_PAN_ lineages in the Americas and Brazil described in previous studies (Gilbert et al., 2007; Cabello et al., 2014; Mir et al., 2015; Worobey et al., 2016; Arantes et al., 2019; Bello et al., 2019). Finally, the lack of epidemiological data regarding the mode of transmission of individuals analyzed reduced the power of our study to confirm any association between the inferred rate of viral spread and the ecological characteristics of local transmission networks in Amazonas.

In summary, this study highlights that the HIV-1 epidemic in the Amazonas state mostly results from the local expansion of one B_CAR_ strain (B_CAR–BR–I_) introduced around the late 1970s and of multiple B_PAN_ viral strains introduced since the late 1980s. Albeit the earlier introduction of the B_CAR–BR–I_ clade granted a much prolonged period of local spread than that of the B_PAN_ strains, this was compensated by a much higher number of independent introductions and the concurrent establishment of multiple B_PAN_ local transmission networks. Despite significant differences in the pattern of early establishment, major B_CAR_ and B_PAN_ clades circulating in Amazonas have been spreading at a quite similar rate over the last two decades, arguing against the hypothesis of significant differences in their intrinsic transmissibility. Our analyses also demonstrate that major Amazonian B_CAR_ and B_PAN_ clades continued to spread and showed no clear signs of recent epidemic stabilization, supporting the relevance of designing more effective strategies to prevent HIV transmission in the region.

## Data Availability Statement

Publicly available datasets were analyzed in this study. This data can be found here: GenBank accession numbers: KEXV01000001 to KEXV01046877; HQ127524 to HQ127607; KU762066 to KU762066; MH673055 to MH673280; and MW545333 to MW545424; Los Alamos HIV Sequence Database (http://www.hiv.lanl.gov).

## Ethics Statement

Ethical review and approval were not required for the study on human participants in accordance with the Local Legislation and Institutional Requirements. Written informed consent for participation was not required for this study in accordance with the National Legislation and the Institutional Requirements.

## Author Contributions

GB conceived and designed the study and supervised the experiments. IA conducted the experiments and analyzed the data. YO, TG, and MG provided HIV-1 sequence data and intellectual input. IA and GB wrote the first draft of the manuscript. All authors assisted with the writing and approved the final manuscript.

## Conflict of Interest

The authors declare that the research was conducted in the absence of any commercial or financial relationships that could be construed as a potential conflict of interest.

## Publisher’s Note

All claims expressed in this article are solely those of the authors and do not necessarily represent those of their affiliated organizations, or those of the publisher, the editors and the reviewers. Any product that may be evaluated in this article, or claim that may be made by its manufacturer, is not guaranteed or endorsed by the publisher.

## Footnotes

1
http://www.hiv.lanl.gov

2
https://www.ncbi.nlm.nih.gov/genbank/

3
www.ncbi.nlm.nih.gov/BLAST
