# Supplementary material for: Dissemination Dynamics of HIV-1 Subtype B Pandemic and Non-pandemic Lineages Circulating in Amazonas, Brazil
Source: Front Microbiol. 2022 Mar 7;13:835443. doi: 10.3389/fmicb.2022.835443 (PMC8940292; doi:10.3389/fmicb.2022.835443)
Supplement: Supplementary file 1 [file Table_1.DOCX]

**Dissemination dynamics of HIV-1 subtype B pandemic and non-pandemic lineages circulating in Amazonas, Brazil**

Ighor Arantes^1^, Tiago Gräf^2^, Paula Andrade^1^, Yury Oliveira Chaves^3^, Monick Lindenmeyer Guimarães^1^, and Gonzalo Bello^1*^

**1.** Laboratório de AIDS e Imunologia Molecular, Instituto Oswaldo Cruz, Instituto Oswaldo Cruz, Fundação Oswaldo Cruz (FIOCRUZ), Rio de Janeiro, Brasil

**2.** Instituto Gonçalo Moniz, Fundação Oswaldo Cruz (FIOCRUZ), Salvador, Brasil

**3.** Laboratório de Diagnóstico e Controle de Doenças Infecciosas na Amazônia, Instituto Leônidas e Maria Deane, Fundação Oswaldo Cruz (FIOCRUZ), Manaus, Brasil

* Correspondence:

gbello@ioc.fiocruz.br


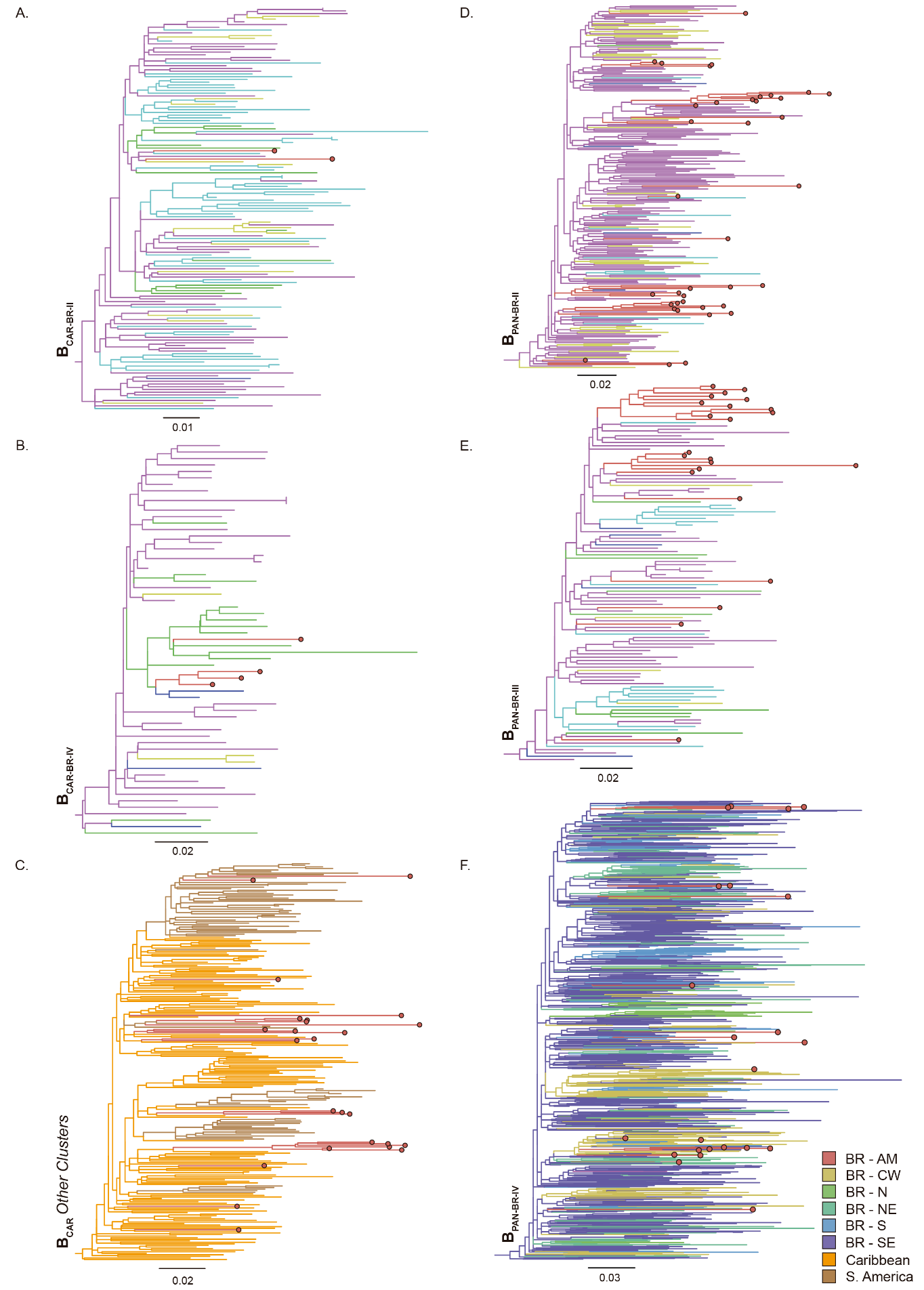


**Figure S1. ML phylogeographic analysis of Amazonian HIV-1 subtype B sequences distributed across major Brazilian clusters and other Caribbean sequences.** The trees represented displayed the B_CAR_ Amazonian sequences that branched within clade B_CAR-BR-II_ (**A**, *n* = 147), clade B_CAR-BR-IV_ (**B**, *n* = 61) or outside major Brazilian clusters (**C,** *n* = 252), and B_PAN_ Amazonian sequences that branched within clades B_PAN-BR-II_ (**D,** *n* = 248), B_PAN-BR-III_ (**E**, *n* = 114), and B_PAN-BR-IV_ (**F**, *n* = 804). The location of taxonomic units at internal nodes across the ML trees was reconstructed and represented according to the color scheme in the bottom right corner. Outside Amazonas state, other units were aggregated according to geographical region. BR: Brazil, AM: Amazonas, CW: central-west, N: north, NE: northeast, S: south, SE: southeast, S. America: South America. Amazonian sequences are indicated with circles. The trees were rooted using HIV-1 subtype D reference sequences (not shown). The branch lengths are drawn to scale with the bar at the bottom indicating nucleotide substitutions per site.


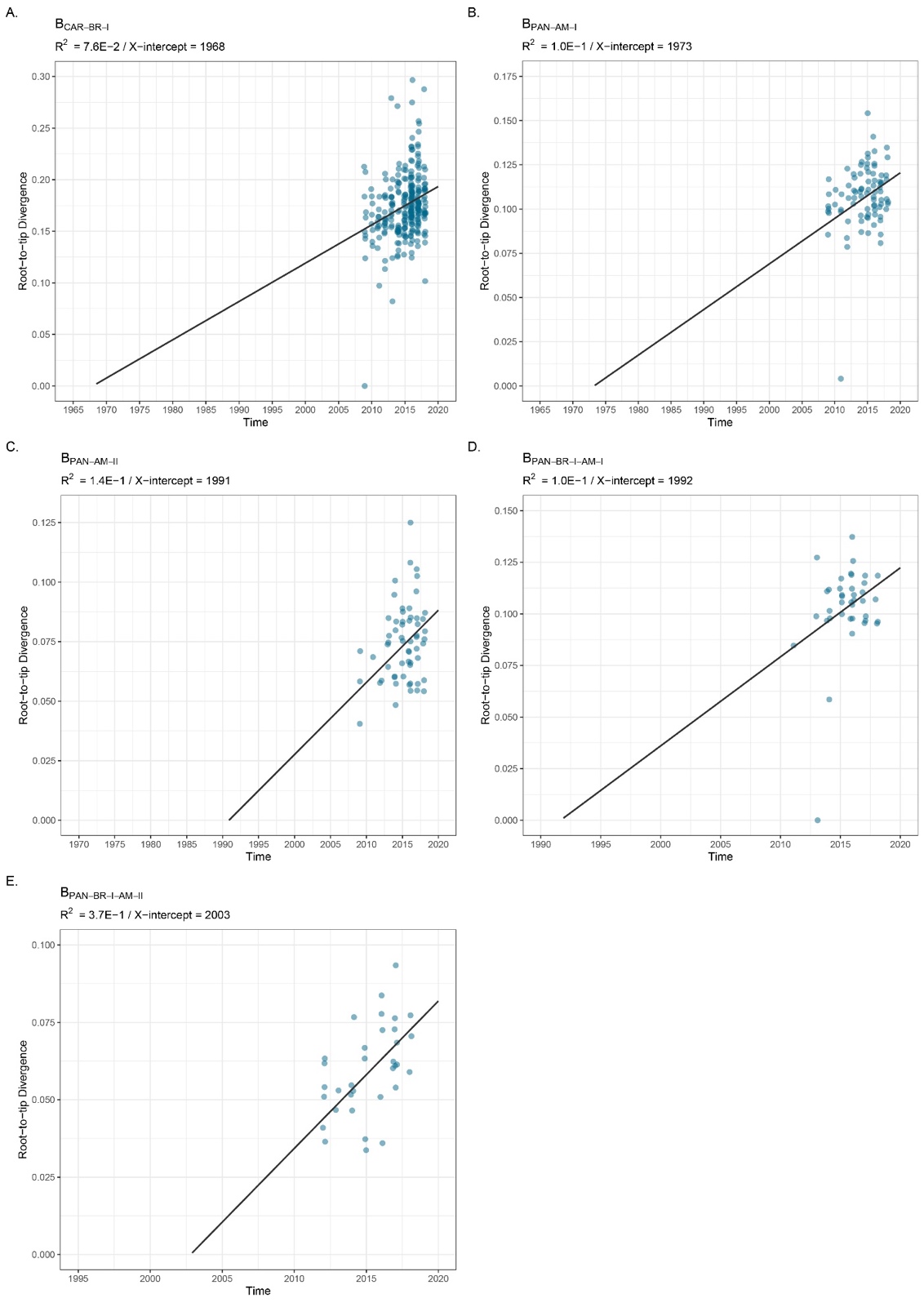


**Figure S2**: **Analysis of the temporal structure of Amazonian HIV-1 subtype B *pol* datasets**. The graphs depict linear regressions of the root-to-tip divergence against sampling date observed in the sequences composing the Amazonian clusters **A)** B_CAR-BR-I_, **B)** B_PAN-AM-I_, **C)** B_PAN-AM-II_, **D)** B_PAN-BR-I-AM-I_, and **E)** B_PAN-BR-II-AM-II_. The ‘best-fitting’ root for the phylogeny was found by maximizing the R^2^ value of the root-to-tip regression. The X-intercept and the R^2^ value is indicated at each graph.

**SUPPLEMENTARY TABLES**

**Table S1. Estimated number of subtype B introductions and size of local clusters in the Amazonas state.**

| **Cluster** | | **Total**  **(N)** | **LC**  **(N/N%)** | **MC**  **(N/N%)** | **SC**  **(N/N%)** | **Singletons**  **(N/N%)** |
| --- | --- | --- | --- | --- | --- | --- |
| B_PAN_ | B_BR-I_ | 100  (360) | 2  (74/21%) | 7  (95/26%) | 39  (139/39%) | 52  (52/14%) |
|  | B_BR-II_ | 15  (38) | - | - | 6  (29/76%) | 9  (9/24%) |
|  | B_BR-III_ | 8  (23) | - | 1  (11/48%) | 1  (6/26%) | 6  (6/26%) |
|  | B_BR-IV_ | 10  (23) | - | 1  (10/43%) | 2  (6/26%) | 7  (7/30%) |
|  | Other | 158  (530) | 2  (146/28%) | 6  (118/22%) | 45  (161/30%) | 105  (105/20%) |
|  | **Total** | **291**  **(974)** | **4**  **(220/23%)** | **15**  **(234/24%)** | **93**  **(341/35%)** | **179**  **(179/18%)** |
| B_CAR_ | B_CAR-BR-I_ | 1  (267) | 1  (267/100%) | - | - | - |
|  | B_CAR-BR-II_ | 2  (2) | - | - | - | 2  (2/100%) |
|  | B_CAR-BR-IV_ | 2  (4) | - | - | 521  (3/75%) | 1  (1/25%) |
|  | Other | 13  (25) | - | - | 5  (17/68%) | 8  (8/32%) |
|  | **Total** | **18**  **(298)** | **1**  **(267/89%)** | **-** | **6**  **(20/7%)** | **11**  **(11/4%)** |

The table details the estimated number of HIV-1 subtype B introductions into the Amazonas state for different variants and the dimension of the clusters derived from them, aggregated in large clusters (LC, *n* > 30), medium clusters (MC, *n* = 10 - 30), small cluster (SC, *n* = 2 - 9), and singletons (*n* = 1). In each group, the number of introductions that expanded to clusters of that particular category, the number of sequences and the fraction it represents in the cluster is shown. Clusters and singletons not nested in any of the four major clusters in either the B_PAN_ or the B_CAR_ forms are collectively referred as “Other”.

**Table S2. Demographic parameters of individuals infected by major HIV-1 subtype B clades originated in the Amazonas state.**

| **Epidemiological Parameter** | | **B_CAR_** | **B_PAN_** | ***P*** |
| --- | --- | --- | --- | --- |
| Age Group | 15 to 24 | 0.09 (19) | 0.09 (11) | 0.90 |
|  | 25 to 39 | 0.56 (114) | 0.58 (74) |  |
|  | 40 to 59 | 0.35 (71) | 0.33 (42) |  |
| Gender | Male | 0.68 (155) | 0.73 (110) | 0.26 |
|  | Female | 0.32 (74) | 0.27 (40) |  |

The table details the distribution of individuals with known information regarding gender and age across major B_PAN_ (*n* = 4) and B_CAR_ (*n* = 4) clusters circulating in the Amazonas state. Distribution of samples across the categories is presented according to their relative frequency followed by the absolute number in parentheses. Samples without information in a particular parameter were omitted and removed from the relative frequency calculation in that parameter. In the “Age Group'' parameter, individuals under 15 years old were omitted due to the vertical transmission associated with infections in this group. Statistical significance was considered under *P* < 0.05.
